# Brain barriers and brain fluid research in 2016: advances, challenges and controversies

**DOI:** 10.1186/s12987-017-0052-7

**Published:** 2017-02-02

**Authors:** Richard F. Keep, Hazel C. Jones, Lester R. Drewes

**Affiliations:** 1 Department of Neurosurgery, University of Michigan, R5018 BSRB, 109 Zina Pitcher Place, Ann Arbor, MI 48109-2200 USA; 2 Gagle Brook House, Chesterton, Bicester, OX26 1UF UK; 3 Department of Biomedical Sciences, University of Minnesota Medical School Duluth, Duluth, MN 55812 USA

## Abstract

This editorial highlights some of the advances that occurred in relation to brain barriers and brain fluid research in 2016. It also aims to raise some of the attendant controversies and challenges in such research.

## Editorial

There continues to be a tremendous interest in brain barriers and brain fluids research. Thus, for example, in 2016 PubMed cites ~4000 papers on CSF, ~2950 papers on the blood–brain barrier (BBB)/neurovascular unit (NVU) and ~1200 papers on hydrocephalus. The purpose of this editorial is to highlight some of the advances made in 2016, as well as some of the attendant controversies. With regards to the latter, we also wish to highlight some of the challenges in such research. With the amount of research published in 2016, it is impossible to cover the breadth of important advances. The choices may seem idiosyncratic but hopefully useful.

## Crossing the blood–brain barrier

One major aim of NVU/BBB research is to develop methods to improve the delivery of therapeutics to the brain. In that regard, there have been several advances in 2016. Deverman et al. used Cre recombination-based adeno-associated virus (AAV) targeted evolution (CREATE) to develop AAV capsids that better target the CNS [1]. That approach resulted in the generation of an AAV variant that widely transfected the mouse brain (neurons and astrocytes) after intravenous injection. The overall CREATE approach may greatly enhance gene transfer into the brain parenchyma for a variety of neurological conditions although there are some issues with the loading capacity of AAVs for large genes.

The delivery of proteins, including antibodies, at therapeutic concentrations across the highly impermeable blood–brain interface has been a major impediment in the development of treatments for neurological conditions. It is, therefore, noteworthy that Sevigny et al. [2] using an antibody, aducanumab, that targets aggregated amyloid-β managed to achieve a brain:plasma area under the curve (AUC) of 1.3% and to dose-dependently reduce both brain soluble and insoluble amyloid-β in a mouse Alzheimer’s disease model. In addition, in a phase 1b clinical trial, aducanumab reduced brain amyloid-β and slowed clinical deterioration in Alzheimer’s patients.

The use of focused ultrasound in conjunction with intravenously administered microbubbles to disrupt endothelial cell tight junctions and enhance blood–brain permeability of therapeutics has been the focus of multiple groups around the world (e.g. [3–5]). The feasibility of this approach has now been shown in non-human primates [5]. Focused ultrasound to enhance drug delivery is now in clinical trial for brain cancers **(**NCT02253212 and NCT02343991). Considerable effort has been made in such studies to determine the safety window for the ultrasound. A concern is that the safety window may vary if the cerebrovasculature is impacted by a neurological condition (e.g. stroke), particularly as that impact may not be homogeneous. Interestingly, Cho et al. also found that focused ultrasound with microbubbles locally down-regulated brain endothelial cell P-glycoprotein expression in rats [6]. This suggests that focused ultrasound can potentially impact brain drug delivery by both inducing structural changes and altering drug transport mechanisms. It does, however, raise the question as to what other endothelial properties, including gene expression, may be affected by the ultrasound. Overall, much effort is being directed towards enhancing delivery of therapeutics to the brain using multiple approaches. The readership is referred to a recent volume of *Drug Discov Today Technol* [7] on some of those approaches.

## Fluid flow within the brain

The concept of a glymphatic system was first proposed by Iliff et al. [8]. It is proposed that there is a rapid movement of CSF from the subarachnoid space into brain along the artery/arteriole paravascular space (linked to arterial pulsation), a movement of fluid from that space through the brain parenchyma where astrocyte aquaporin4 plays an important role, and a return of fluid to subarachnoid space along the paravascular space of venules/veins. This circulation may provide a way of clearing potentially toxic waste products, such as amyloid-β, from the brain. Since its first proposal, the concept of a glymphatic system has gained great traction with ~40 papers addressing glymphatics in 2016 alone (PubMed) and there have been excellent reviews on the subject [9–11]. It has been the spur for innovative research, including the impact of sleep on the system [12] and even its potential role in regards to the eye [13]. The application of ultrafast MRI techniques has led to the suggestion that in addition to arterial pulsations, low frequency vasomotor activity may provide assistance to glymphatic propulsion through the interstitial space [14] and also has enabled visualization of the perivascular spaces with sub millimeter resolution [15]. It should be noted, however, that there have been recent challenges to the concepts underlying the proposed glymphatic system in particular related to the role of arterial pulsation [16] and the role of astrocyte water permeability/aquaporin4 [17].

There also continues to be some controversy as the relative role of the choroid plexus in CSF production. The readership is referred to a number of recent reviews addressing that subject [9, 18–22]. In models of CSF flow there is generally a vectoral component (e.g. through the ventricular system), although there can be a large oscillation around that flow based on the respiratory and cardiac cycles [23]. However, recent evidence shows that within the ventricular system there are complex flow patterns generated by cilia networks [24]. Interestingly, those flow patterns change not only spatially, but also temporally. These flow patterns may be important in regulating communication between different periventricular areas.

A related question to how much fluid is produced by the choroid plexus is how much fluid is produced across the cerebral capillaries, by what mechanisms, and how is fluid production affected by disease states? Fluid production and ion transport by cells of the NVU have been dealt with in depth in a recent review by Hladky and Barrand [19]. While such fluid transport cannot result from filtration across capillaries and needs to be linked to ion transport, defining the precise mechanisms involved (and how to manipulate them) has proven very difficult [19]. Studies of brain endothelial transport in vivo are complicated by the presence of other components of the NVU. Perhaps progress can be made on vectoral ion and fluid transport with the newer generation of in vitro models with higher transendothelial electrical resistance (TEER) and lower paracellular permeability (see below). Choroid plexus epithelial cell cultures with high TEERs have been used to demonstrate fluid transport [25]. An understanding of NVU/BBB fluid transport in disease states is vital for developing methods to reduce brain edema formation [26]. Methods of treating cerebral edema have not changed in decades.

## Choroid plexus

Apart from fluid secretion and ion transport, there has been an upsurge in interest in other functions of the choroid plexus. Evidence now indicates that it has important functions in immune surveillance [27], in stem cell regulation [28] and in brain signaling via extracellular vesicles [29]. The choroid plexus may also be a potential route for drug delivery into the CSF by transcytosis [30].

## Hydrocephalus

Fetal hydrocephalus can be induced in mice by intracerebral injection of blood or lysophosphatidic acid (LPA) and such hydrocephalus can be blocked by an LPA receptor antagonist [31]. Interestingly, Park et al. [32] have recently investigated the molecular mechanisms underlying LPA-induced hydrocephalus and found that LPA is an upstream regulator of the protein YAP which is important in the development of ependymal junctions. Further dissection of these pathways may give insights into the potential causes of some forms of hydrocephalus.

Although congenital hydrocephalus can have non-genetic causes, such as intraventricular hemorrhage, over 100 genetic causes have now been identified [33]. Several biochemical pathways are implicated including Wnt signaling, cell junction integrity and especially cilia function (e.g. [34, 35]). However, despite our advances in understanding underlying causes, shunt treatment or ventriculoscopy continue to be the only treatment available for hydrocephalus [36]. Despite efforts to improve shunt design, treatment still carries large risks.

Much effort continues to be directed towards idiopathic normal pressure hydrocephalus (iNPH) defined by enlarged ventricles and a triad of clinical signs in older people. Many theories of pathogenesis exist for this complex condition and are comprehensively reviewed by Keong et al. [37]. Problems exist in distinguishing which patients will respond to shunt treatment (e.g. [38, 39]). A multimodal analysis including clinical signs, MRI parameters, intracranial pressure and CSF biomarkers did not predict shunt responders in a cohort of 284 patients [40]. Diffusion tensor imaging (DTI) has been used to determine white matter pathology in hydrocephalus [41] and changes after shunt treatment [42].

## The blood–brain barrier/neurovascular unit in disease

Many neurological conditions are accompanied by BBB dysfunction (e.g. stroke, traumatic brain injury and brain neoplasms) with increased transfer of even proteins from blood to brain. There has always been a question of whether the BBB dysfunction is a secondary result of parenchymal injury or a primary site of injury. Recent results from Shi et al. found that endothelial-specific overexpression of a mutant form of actin depolymerizing factor reduced barrier disruption after stroke and ameliorated acute and chronic brain injury [43]. These results strongly suggest that barrier dysfunction may be a cause rather than just result from parenchymal damage after stroke and that it is an important therapeutic target.

Currently, the only therapeutic intervention for ischemic stroke is thrombolysis-induced reperfusion with tissue plasminogen activator (tPA) and now thrombectomy [44]. One major concern about the use of tPA to induce reperfusion is potential barrier dysfunction and hemorrhagic transformation. There has, therefore, been interest in combining tPA with an agent that protects the cerebrovasculature. Wahlgren et al. [45] conducted a phase II randomized clinical trial combining intravenous thrombolysis with tPA and imatinib, an agent that protects the cells of the NVU and reduces hemorrhagic transformation in animal stroke models [46, 47]. Wahlgren et al. found that imatinib was safe and that it showed some evidence of improved outcome in the stroke patients [45].

One function of brain endothelial cells is to supply essential nutrients to the brain. Thus, those cells express high levels of the glucose transport GLUT 1 (SLC2A1) and the large neutral amino acid transporter LAT1 (SLC7A5). The importance of mutations in GLUT1 have long been known (glucose transporter type 1 deficiency syndrome; [48]) but Tarlungeanu et al. [49] have recently found that homozygous mutations in LAT1 are associated with autistic traits and that endothelial deletion of LAT1 in mice causes severe neurological defects that can be ameliorated by intracerebroventricular administration of branch-chain amino acids.

The use of serum (and CSF) biomarkers for neurological conditions continues to be an area of intense research. For example, one particular focus has been the use of biomarkers for diagnosis and prognosis in different grades of traumatic brain injury; e.g. concussion [50]. A recent article by Dadas et al. [51] in *Fluids Barriers CNS* highlights the multiplicity of factors that impact biomarker levels in blood and presents modeling to enhance interpretation.

The cerebrovasculature has functions outside what are normally considered as ‘barrier’ properties. One such function is to form a vascular niche for neural stem cells promoting self-renewal and inhibiting differentiation [52] and there has been considerable interest in manipulating that niche to target cancer stem cells in glioblastoma and other brain cancers [53]. The cerebrovasculature can, however, also be a route for tumor invasion into surrounding tissue and Yadav et al. recently showed that such invasion can be reduced by targeting a chemokine receptor, CXCR4, improving survival in a mouse glioma model [54].

## In vitro blood–brain barrier/neurovascular unit modeling

Helms et al. recently provided an excellent review of current in vitro NVU/BBB models, their uses and limitations [55]. Progress continues to be made. Of note, Canfield et al. [56] recently showed that induced pluripotent stem cells (iPSCs) from a single patient can be used to derive endothelial cells, astrocytes and neurons and produce a BBB/NVU model with transendothelial electrical resistances and permeabilities close to in vivo. An ultimate goal for a number of groups is to produce microfluidic devices, a ‘BBB on a chip’ (e.g. [57–60]). An advantage of those models, as well some other larger scale models, is the incorporation of shear stress, an important determinant of endothelial characteristics. Big strides are being made in developing in vitro NVU/BBB models with high TEERs and low passive permeabilities to hydrophilic molecules. A remaining challenge is whether those models can also recapitulate other NVU/BBB characteristics; e.g. relevant expression/activity of transporters and the metabolizing enzymes that form the metabolic barrier, as well as rates of transcytosis.

## The future

As can be seen from this brief summary of just some of the research into the brain barriers and brain fluids, major strides have been made in 2016. It is a vibrant area of research and one that is always associated with areas of controversy. Technical advances are being made that will hopefully address those controversies and open new avenues for research.
